# Characteristics of ultrasonographic images of ductal carcinoma in situ with abnormalities of the ducts

**DOI:** 10.1007/s10396-019-00981-z

**Published:** 2019-10-26

**Authors:** Kanako Ban, Hiroko Tsunoda, Takanori Watanabe, Setsuko Kaoku, Takuhiro Yamaguchi, Ei Ueno, Koichi Hirokaga, Kumiko Tanaka

**Affiliations:** 1 Department of Cancer Detection and Diagnosis, Tokyo Health Service Association, 1-2 Ichigaya-Sadohara-cho, Shinjuku-ku, Tokyo, 162-8402 Japan; 2 Department of Radiology Diagnostic Breast Imaging, St. Luke’s International Hospital, Chuo-ku, Tokyo, Japan; 3 Department of Breast Surgery, National Hospital Organization Sendai Medical Center, Sendai, Miyagi Japan; 4 National Hospital Organization Osaka National Hospital, Osaka, Japan; 5 Clinical Research Data Center, Tohoku University Hospital, Sendai, Miyagi Japan; 6 Tsukuba International Breast Clinic, Tsukuba, Ibaraki Japan; 7 Department of Breast Surgery, Hyogo Cancer Center, Akashi, Hyogo Japan; 8 Department of Breast Surgery, Shonan Kamakura General Hospital, Kamakura, Kanagawa Japan

**Keywords:** Breast cancer, Ultrasonography, DCIS, Non-mass abnormalities, Duct abnormalities

## Abstract

**Purpose:**

Although the number of ductal carcinoma in situ (DCIS) cases has increased with the spread of breast cancer screening in Japan, there are very few reports that summarize ultrasound image features of DCIS. The Japan Association of Breast and Thyroid Sonology (JABTS) investigated the incidence of DCIS with masses and non-mass abnormalities and the characteristics of US images in a retrospective, multicenter, observational study (JABTS BC-02 study). The purpose of this report is to clarify the proportion of DCIS with abnormalities of the ducts with each ultrasound finding and the characteristics of US images.

**Methods:**

The JABTS BC-02 study population was comprised of patients who were examined by ultrasonography, underwent surgery, and were histopathologically diagnosed with DCIS at each study site between January 2008 and December 2012. The US images of DCIS and pathology and clinical information were retrospectively collected from 16 institutions in Japan. The US images were evaluated by 22 experts on the Central Image Interpretation Committee of JABTS.

**Results:**

Abnormalities of the ducts were noted in 78 (10.5%) of 705 US images of DCIS. Of the 78 cases, the distribution of abnormalities of the ducts was focal or segmental. The second characteristic was the presence of internal echoes in dilated ducts. All cases were accompanied by intraductal solid echoes, and 40 cases (51.3%) were accompanied by echogenic foci. In addition, intraductal solid echoes were continuous or multiple in 72 cases (92.4%), and the shape of the solid echoes was broad-based and/or irregular in 62 cases (79.5%).

**Conclusion:**

DCIS cases with duct abnormalities on ultrasound were investigated in this study. The important characteristics were as follows: (1) the distribution of ductal dilatation was focal or segmental, (2) solid parts were present in the dilated ducts, (3) the distribution of internal echoes was continuous or multiple, (4) the shape of solid echoes was broad-based and/or irregular, and (5) internal echoes were sometimes accompanied by echogenic foci. Accurate evaluation of these findings may be useful for diagnosing DCIS. Although the duct abnormalities are included in “ASSOCIATED FEATURES” in ACR BI-RADS ATLAS (USA), we emphasize that this concept is very important for understanding US characteristics of DCIS.

## Introduction

In Japan, breast cancer screening by mammography (MG) has been performed since 2000 for females aged 50 years or older, and since 2004 for females aged 40 years or older, increasing the detection rate of ductal carcinoma in situ (DCIS). When the necessity of further examination is indicated by MG screening, ultrasonography (US) is performed as a detailed examination in many cases. In Japan, breast cancer screening by US is also performed at many private healthcare organizations. Therefore, it is important to understand the US features of DCIS. The characteristics of US features of DCIS have been investigated in a single institution before, but reports of multicenter studies have not been published. Therefore, the Japan Association of Breast and Thyroid Sonology (JABTS) performed a multicenter, retrospective, observational study (JABTS BC-02 study) of the characteristics of DCIS on US. JABTS classifies breast US findings into masses or non-mass abnormalities in the Japanese guideline third edition published in 2014 [1]. It has been reported that many DCIS cases are evaluated as masses and non-mass abnormalities on US images [2]. Non-mass abnormalities are further classified into five subtypes: abnormalities of the ducts, hypoechoic area in the mammary gland, architectural distortion, clustered microcysts, and echogenic foci without hypoechoic areas.

There are some DCIS cases found with abnormalities of the ducts, but there have been few articles clearly describing the ultrasound findings of these DCIS. The purpose of this report is to clarify the proportion of DCIS with abnormalities of the ducts with each ultrasound finding and the characteristics of US images in a retrospective manner.

## Subjects and methods

The subjects in the JABTS BC-02 study were patients who were examined by ultrasonography, underwent surgery, and were histopathologically diagnosed with DCIS at each study site between January 2008 and December 2012. The US images of DCIS and pathology and clinical information were retrospectively collected from 16 institutions in Japan. In the BC-02 study, 809 cases of DCIS were investigated. Excluding 104 cases in which only calcifications on MG were noted without abnormal US findings, 705 cases had confirmed US findings. In this study, only B-mode data were used. For this reason, this study was a multicenter study and various devices were used. The elastography devices, in particular, differed greatly from one device to another. Criteria for evaluation of non-mass abnormalities, especially duct abnormalities, have not been established for elastography or even color Doppler. The US images were evaluated by 22 experts on the Central Image Interpretation Committee of JABTS. The 22 experts were doctors and sonographers on the JABTS Terminology and Diagnostic Criteria Committee, were qualified as Fellow of the Japan Society of Ultrasonics in Medicine (JSUM) or JSUM Registered Medical Sonographer, and had sufficient breast ultrasound skills. When opinions on the evaluation were divided, the final decision was made after consultation with all members on the Central Image Interpretation Committee.

The subjects in this study included not only breast cancer screening cases, but also medical cases. The symptoms and other factors behind detection are listed below as background factors: MG screening in 28 cases, US screening in 11 cases, based on subjective symptoms in 23 (nipple discharge in 15, palpation of a mass in 8), screening by palpation in 5 (nipple discharge in 3, palpation of a mass in 2), combination of MG and US screening in 1, and others in 9, including detection during follow-up after surgery for breast cancer in 5, detection by further examination of other findings in 3, and incidental detection on PETCT in 1 (some cases had more than one factor).

Since this study was a multicenter study that looked at data from 16 sites, various ultrasonic instruments were used, but all the sites used full digital ultrasound equipment and high-frequency probes. Information on all the devices is shown in Table 1.

**Table 1:** List of equipments

ALOKA SSD 5500 (ALOKA) + B2:B16
ALOKA F75,α7 (ALOKA)
Aplio 50,80,300,400,500 (TOSHIBA)
Aplio MX SSA-780A (TOSHIBA)
Aplio XG SSA-770A,790A,700A (TOSHIBA)
HI VISION Ascendus (HITACHI)
HI VISION Avius (HITACHI)
HI VISION Preirus (HITACHI)
HITACHI EUB 7500,8500 (HITACHI)
prosound α7,α10 (HITACHI)
HD-11XE (Phillips)
HDI 5000 (Phillips)
iU 22 (Phillips)
Logic700MR (GE)
LOGIQ 7,9,e,S6,700MR (GE)
ACUSON S2000 (Siemens)

## Results

There were 705 DCIS cases collected throughout the BC-02 study, of which 260 cases (36.9%) were masses and 428 cases (60.7%) were non-mass abnormalities. In the remaining 17 cases (2.4%), masses and non-mass abnormalities were coexistent to the same degree and could not be classified as one or the other [2]. Details of the findings were as follows: hypoechoic areas in the mammary gland were found to be the most frequent lesion (48.6%) of DCIS lesions, followed by solid masses (28%) and abnormalities of the ducts (10.2%) or mixed masses (8.1%). Architectural distortion (1.3%), clustered microcysts (1.4%), and echogenic foci without a hypoechoic area (2.5%) were rare.

**Fig. 1:**
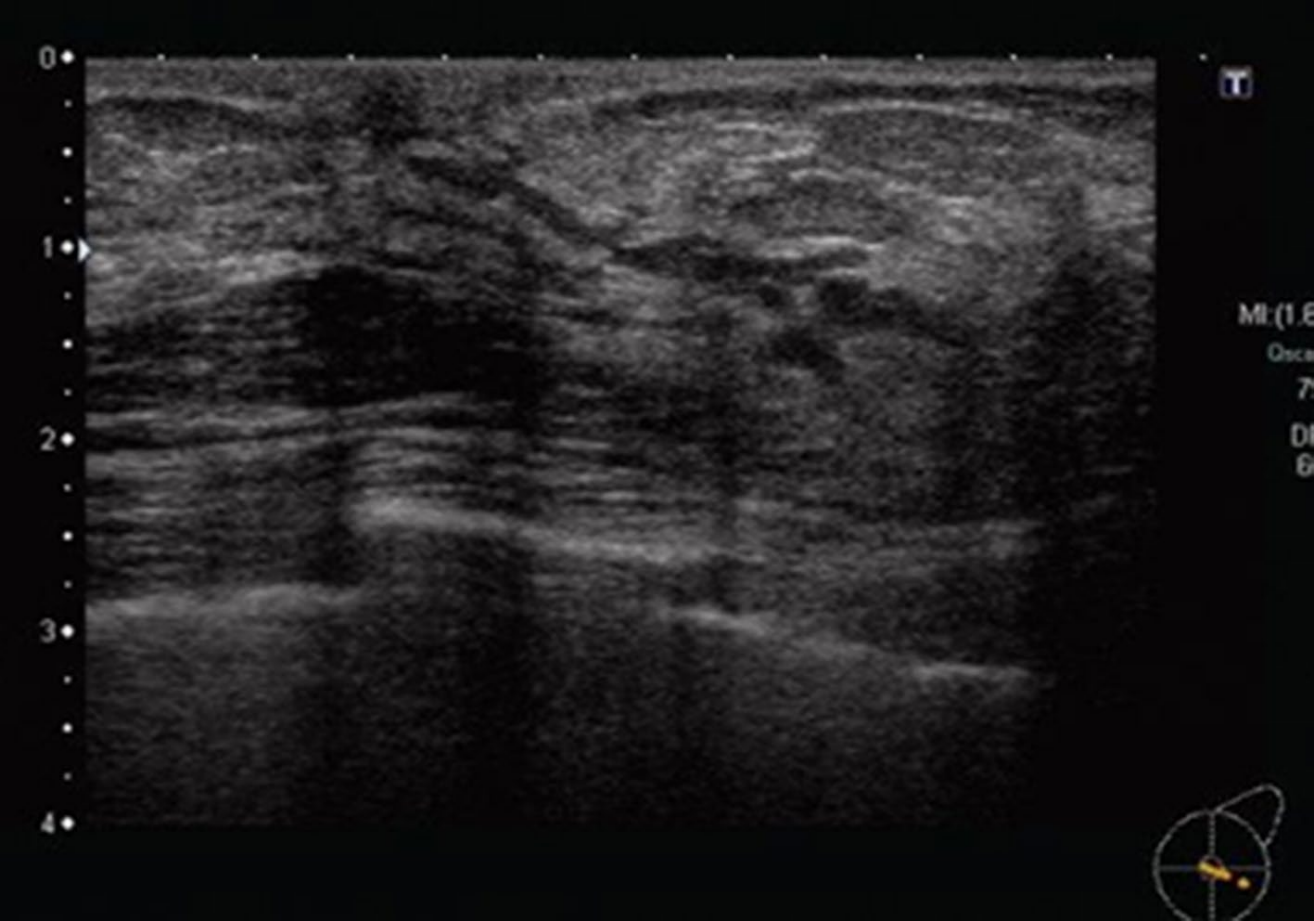
A case with duct abnormalities alone

**Fig. 2:**
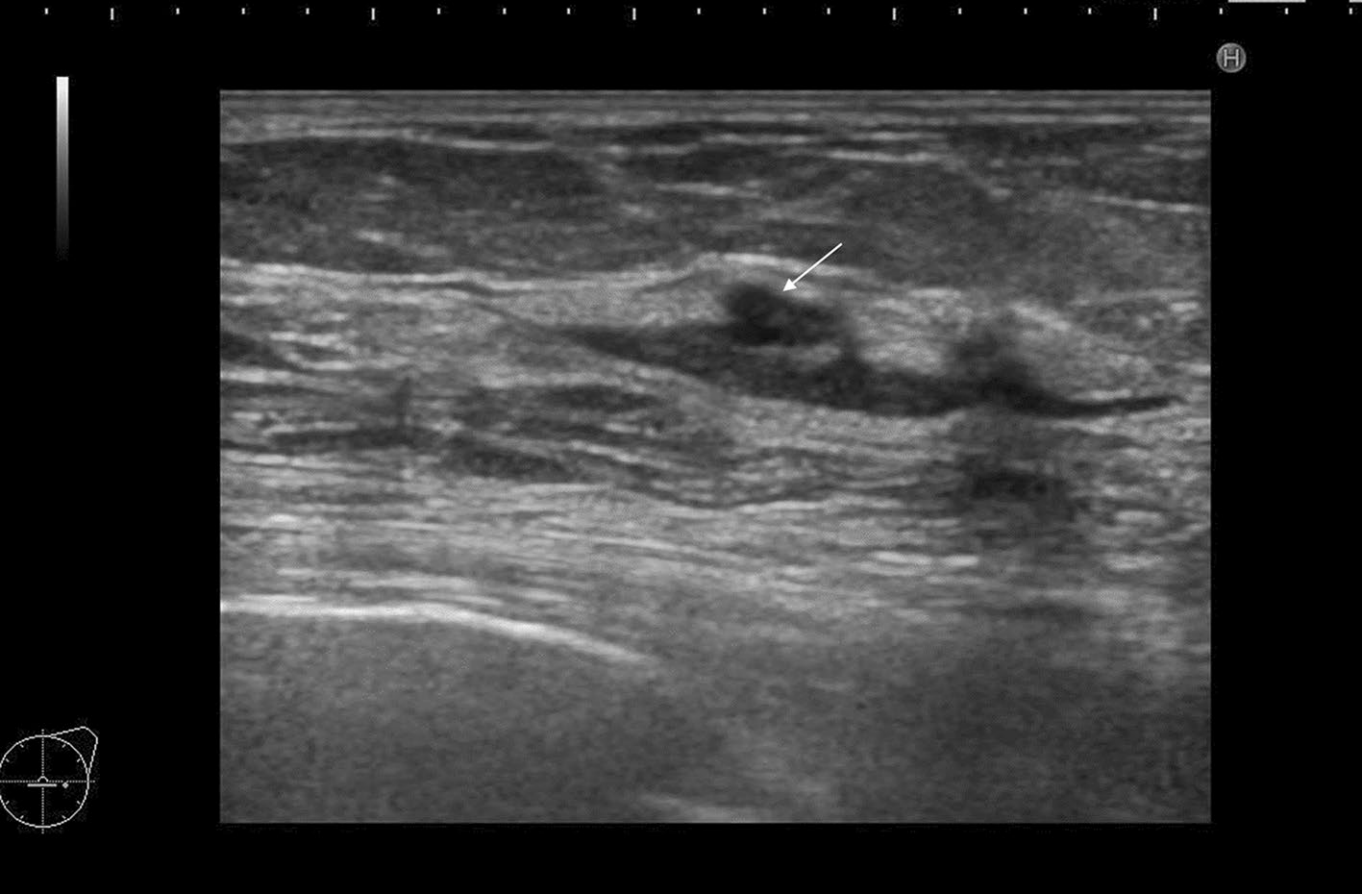
A case with duct abnormalities with a mass

**Fig. 3:**
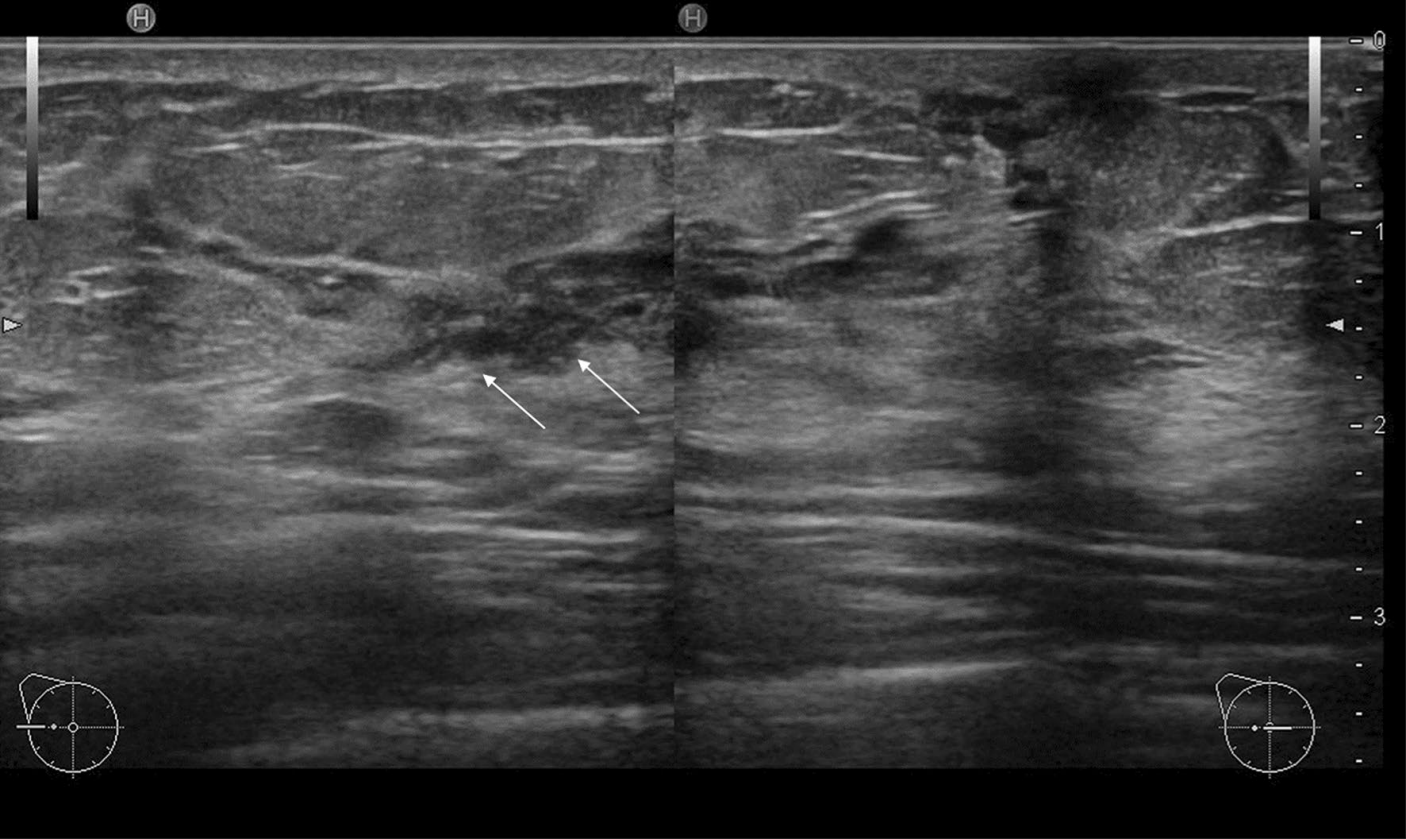
A case with duct abnormalities with hypoechoic areas

The breast ultrasound findings might sometimes be accompanied by secondary findings in addition to the primary findings. In this report, the main findings were breast duct abnormalities, so masses and hypoechoic areas were treated as secondary findings. Therefore, the duct anomalies were classified into three categories, as shown in Table 2: (1) the duct abnormalities alone (Fig. 1), (2) the duct abnormalities with masses (Fig. 2), and (3) the duct abnormalities with hypoechoic areas (Fig. 3). Duct abnormalities alone means the duct abnormalities were not accompanied by secondary findings such as masses and hypoechoic areas. It did not matter whether there were internal echoes in the ducts.

**Table 2:** Duct abnormalities were classified into three categories

1. Duct abnormalities alone
2. Duct abnormalities with masses
3. Duct abnormalities with hypoechoic areas

Of the 78 cases with abnormalities of the ducts, there were 48 cases with duct abnormalities alone (Fig. 1), masses were concomitantly present as part of the lesion in 11 cases (Fig. 2), and hypoechoic areas in the mammary gland were concomitantly noted as part of the lesion in 19 cases (Fig. 3).

The Japanese breast ultrasonography guidelines were published in 2004 to standardize terms and diagnostic criteria. This report followed the guidelines published in 2014 as the latest third edition [1].

**Fig. 4:**
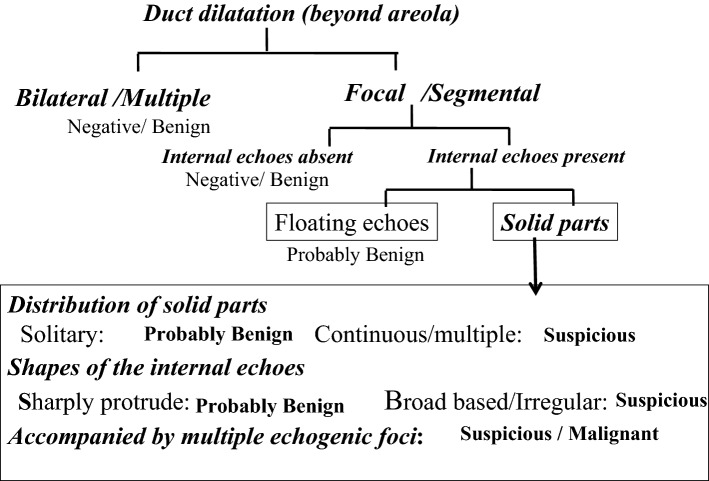
Assessment of abnormalities of the ducts

Figure 4 shows the decision tree for the determination of benign or malignant duct abnormalities according to the Japanese guidelines [1]. Future expressions and terms are explained according to the content and terms of the guidelines.

**Fig. 5:**
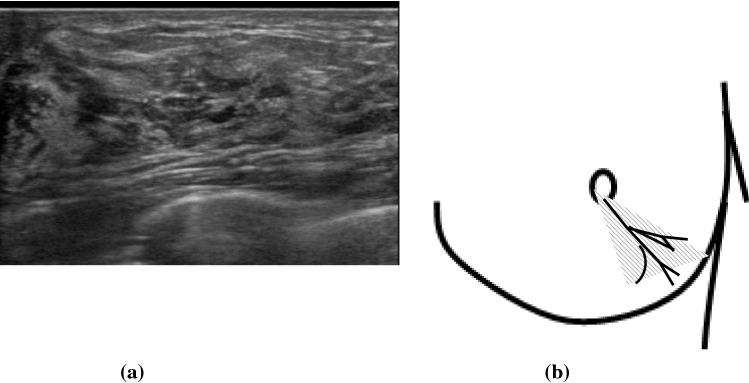
Segmental distribution of duct dilatation (**a**). Schema of the segmental distribution (**b**)

**Fig. 6:**
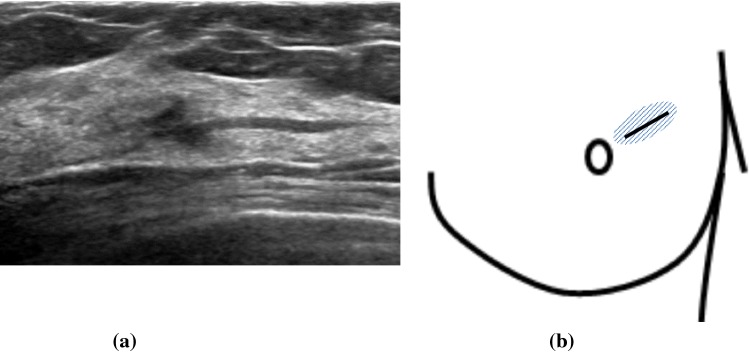
Focal distribution of duct dilatation **a**. Schema of the focal distribution **b**

**Fig. 7:**
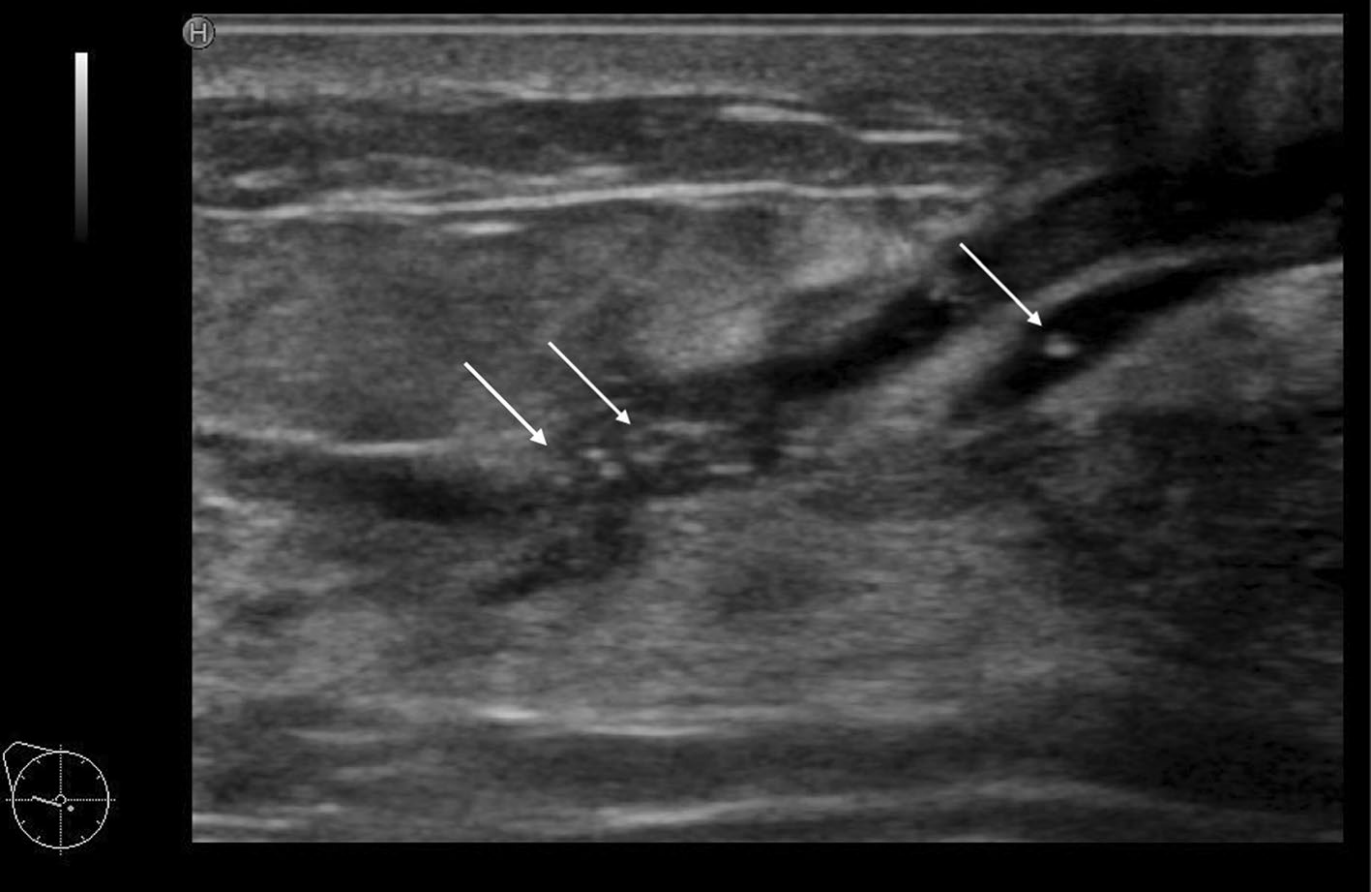
Multiple echogenic foci in the dilated ducts

First, the distribution patterns of the abnormal ducts were reported. All 78 DCIS cases showed focal or segmental distribution. The definition of segmental is a distribution that is found to be consistent with the mammary glandular system, and focal means it is restricted to a certain area. There were 37 cases with segmental distribution (Fig. 5a, b) and 41 cases with focal distribution (Fig. 6a, b). Of these, wall thickening of the duct was clearly observed in one case and irregularity of the duct caliber was noted in ten cases. Furthermore, the internal echoes within the ducts were detected in all 78 cases. Regarding the internal echoes in the ducts, solid echoes were found in all 78 cases (100%), and 40 cases (51.3%) were accompanied by multiple echogenic foci (Fig. 7).

**Fig. 8:**
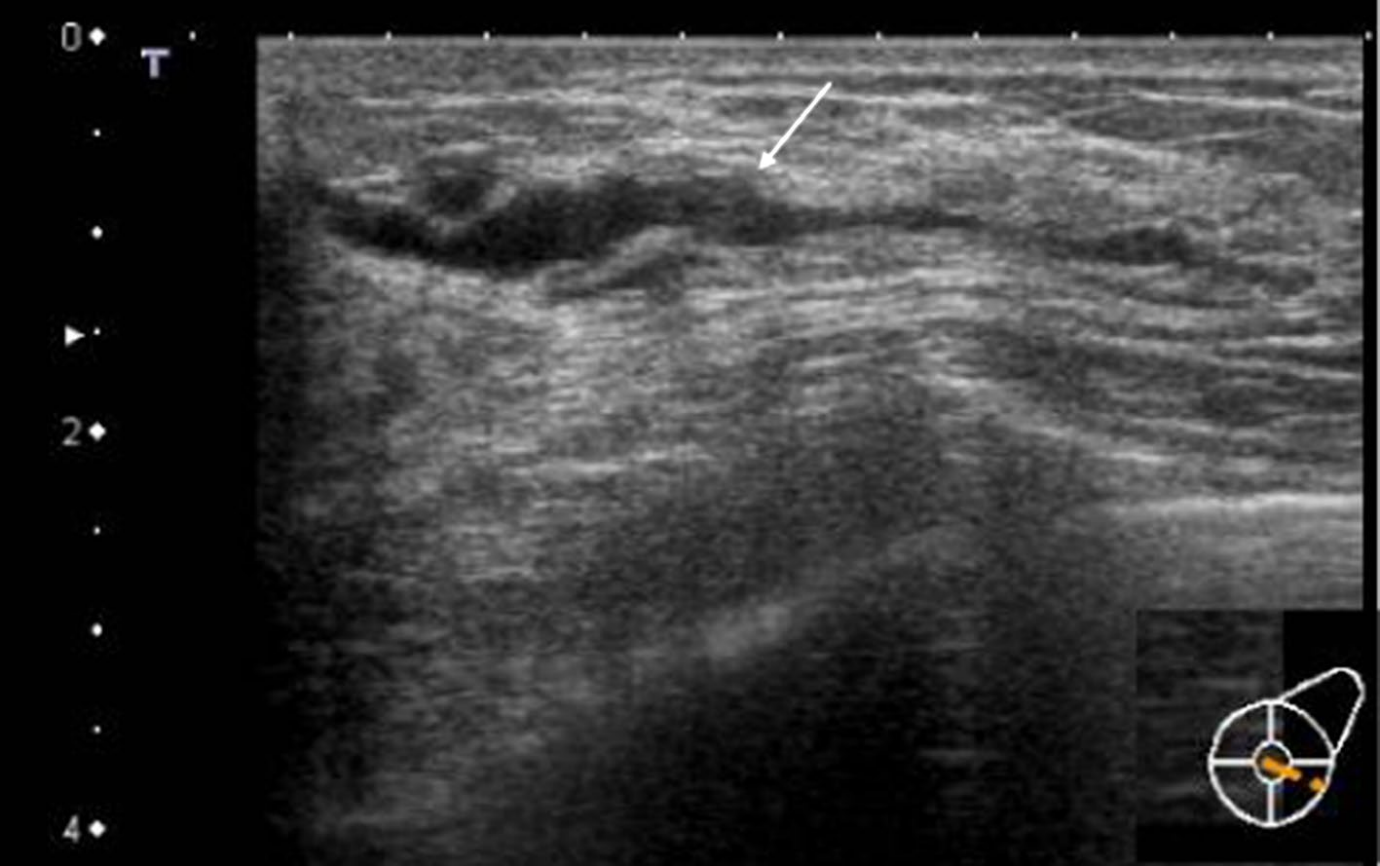
Solitary solid part in the duct

**Fig. 9:**
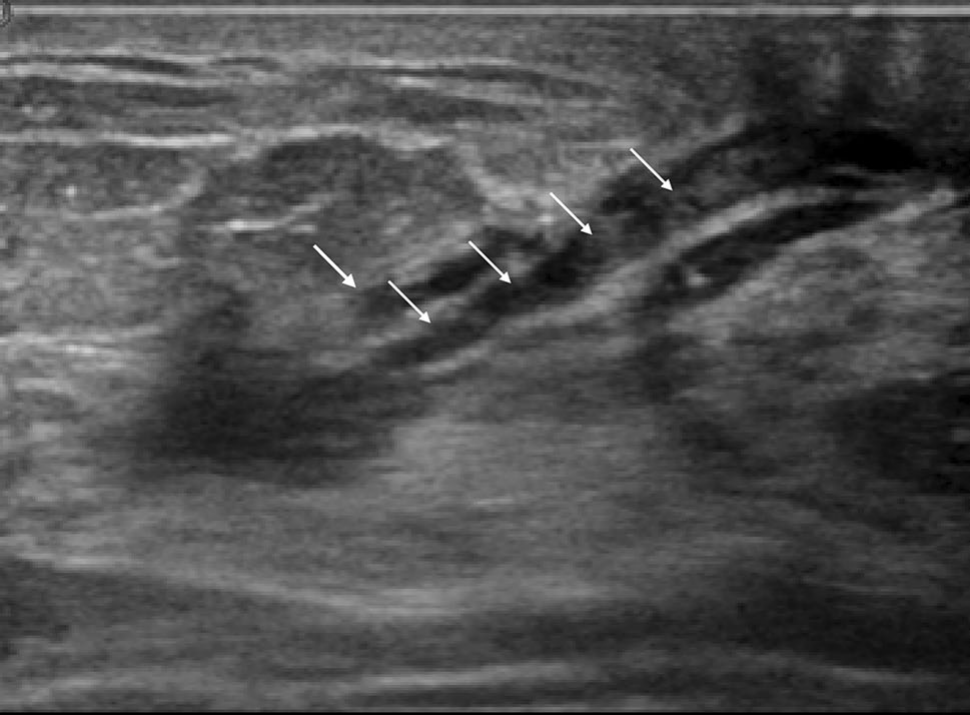
Distribution of continuous solid parts in the ducts

**Fig. 10:**
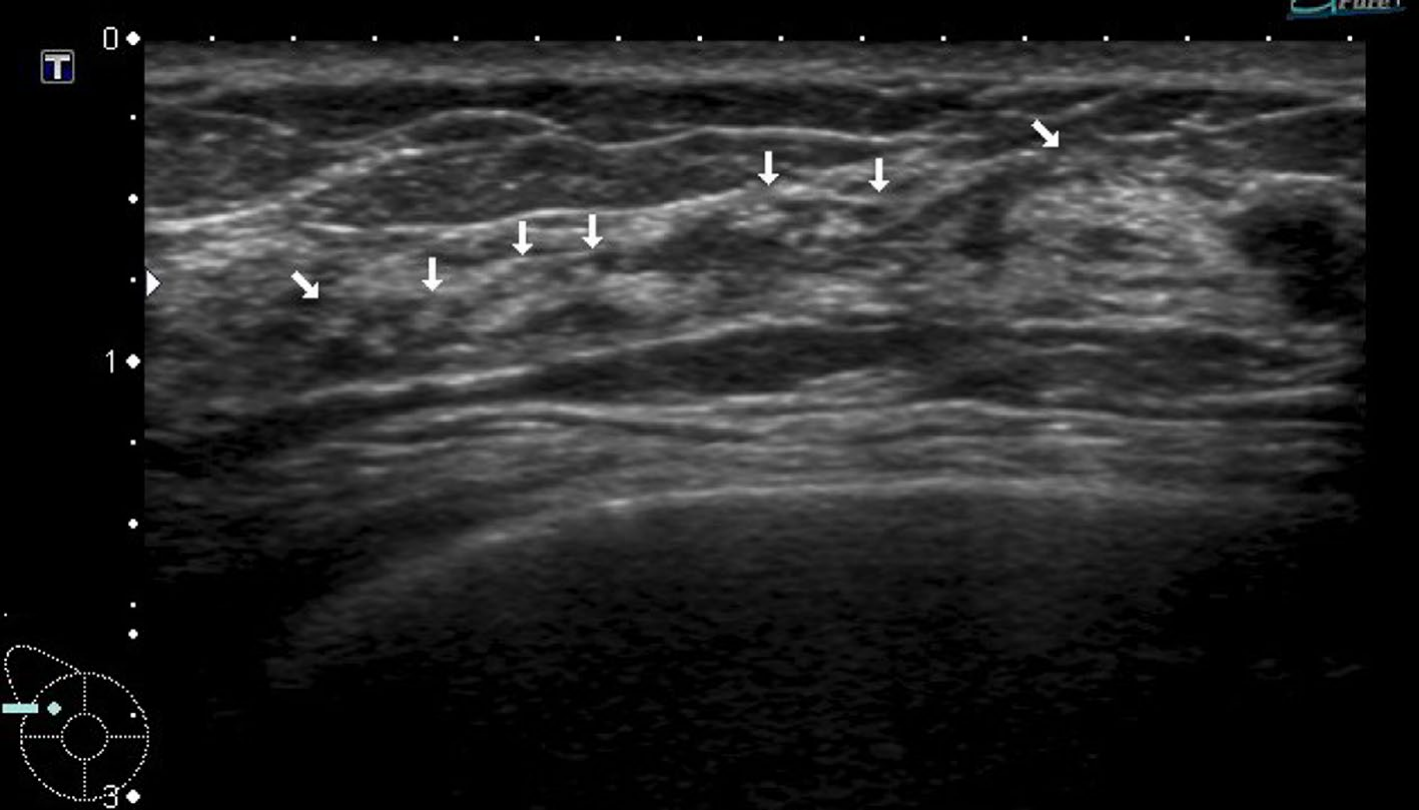
Distribution of multiple solid parts in the ducts

**Fig. 11:**
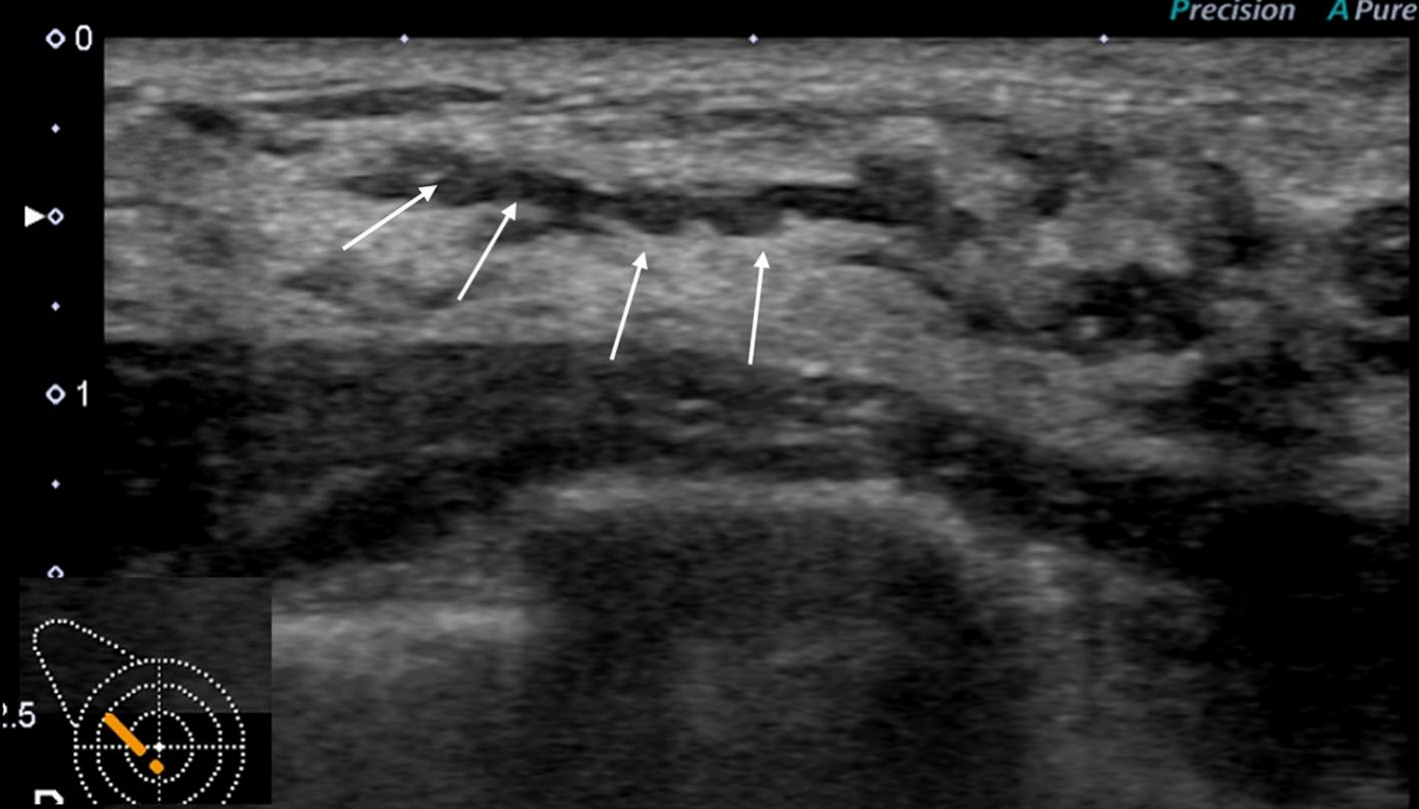
The shape of the solid part is broad-based (or irregular). It is difficult to strictly distinguish between broad-based cases and irregular cases

Next, the features of the distribution and shape of the solid parts were described. Continuous, multiple, broad-based, and irregular, which represent the distribution and shape of the solid part in the ducts described below, are all terms based on the Japanese guidelines (Fig. 3). The distribution of solid echoes was solitary (Fig. 8) in 3 cases (3.8%) and continuous (66)/multiple (6) in 72 cases (92.4%), and evaluation was difficult in 3 cases (3.8%). Continuous distribution is a pattern that has a long continuous range of solid parts in the duct (Fig. 9), and multiple distribution is a pattern that has many single solid parts (Fig. 10). When two or more patterns were mixed, the dominant pattern was described. The shape of the solid part was broad-based (or irregular) in 62 cases (79.5%) (Fig. 11), and it was difficult to express the shape in 16 cases (20.5%). The term ‘broad-based’ may also be expressed as the term ‘irregular’ in the Japanese guideline. It is difficult to strictly distinguish between broad-based cases and irregular cases. There were no cases with sharply protruding solid parts.

## Discussion

In mammography screening, microcalcifications are well known to be one of the characteristic findings of DCIS, but the typical findings of DCIS on breast US have not been clear. That was the reason for conducting the BC-02 study.

JABTS published the Japanese breast ultrasonography guidelines in 2004, and they are currently used for both screening and clinical examinations [1]. JABTS classifies breast US findings into masses and non-mass abnormalities. Ueno et al. divided US images of breast disease into “tumor image-forming type” and “non-tumor image-forming type” [3]. These terms became the source of the current “mass” and “non-mass abnormalities”. Although the concept of “non-mass abnormalities” is thought to be important in Japan, there is no such terminology in ACR BI-RADS ATLAS (USA) [4]. Recently, there are even some reports outside Japan based on the concept of non-mass abnormalities from the United States and Korea [5–7]. In the Japanese guidelines, whether a lesion is a mass or a non-mass abnormality is defined on ultrasonography regardless of whether a mass (lump) is clinically palpated. Abnormalities of the ducts are defined as ‘differences in the thickness, lumen, or wall of the mammary duct from those of normal mammary ducts’. In ACR BI-RADS ATLAS (USA) [4], there is a term similar to abnormalities of the ducts in the Japanese criteria, “abnormalities of the duct”, but this is included in “ASSOCIATED FEATURES” in BI-RADS, in which it is described as follows: there are two concepts, irregular dilatation of a single duct, and dilated ducts with some echogenic intramammary ductal material.

**Fig. 12:**
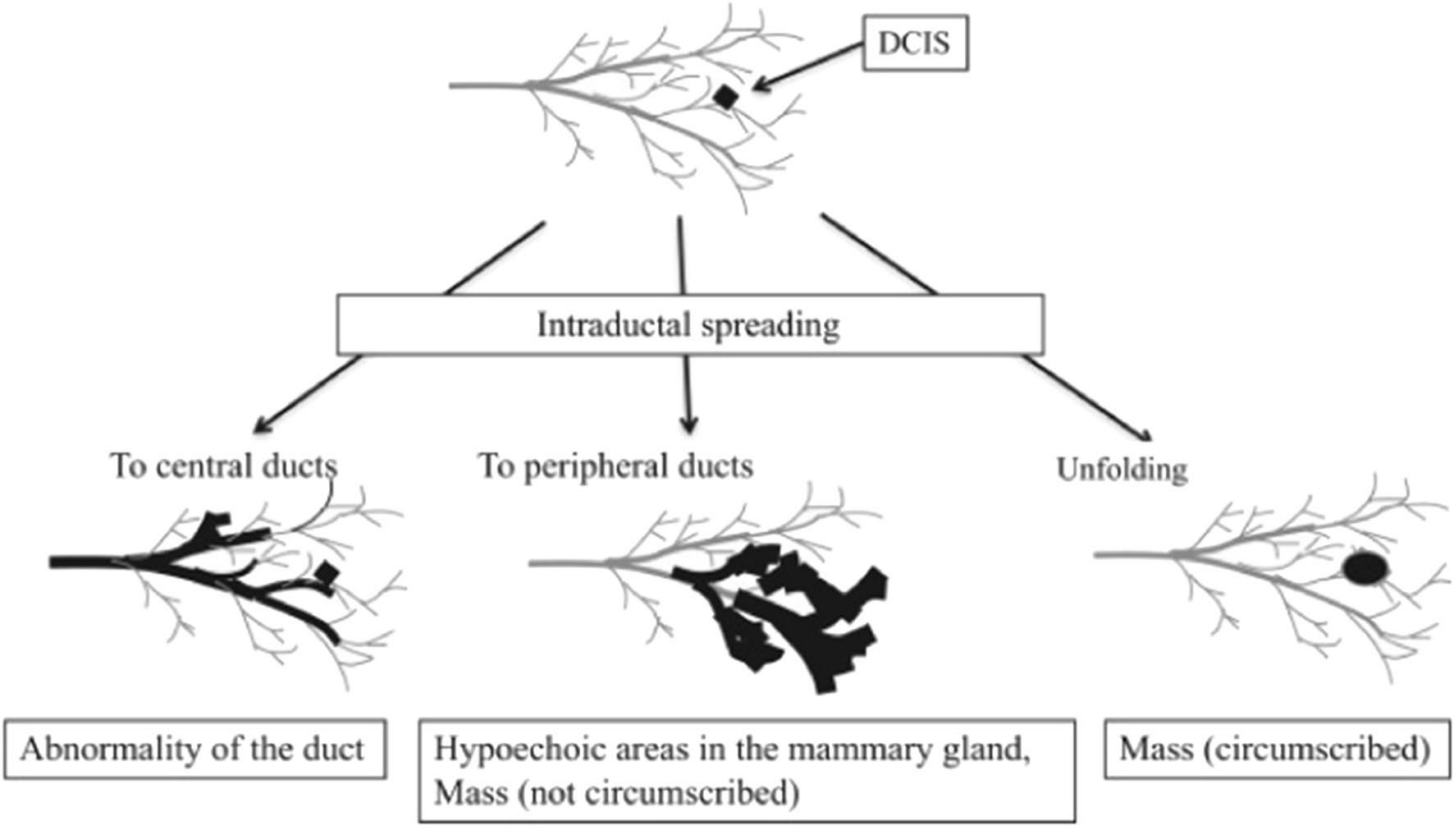
Schematic of our concept of the relationship between ductal carcinoma in situ progression and ultrasound imaging findings. Excerpted from fig. 7 in Reference 2 (Watanabe et al. 2017)

Given the mechanism of occurrence of DCIS, it is very important to understand that duct abnormalities are not “ASSOCIATED FEATURES”, but rather an independent concept [8–12]. Abnormalities of the ducts may be more easily understood by considering the development of breast cancer. Breast cancer develops in the terminal mammary duct lobular unit (TDLU) [6], and there are outgrowth of mammary ductal epithelium and secretions, dilating the duct. This ductal dilatation is regarded as the first finding of breast cancer development among the features of abnormalities of the ducts. Cancer cell proliferation forms a solid part in the dilated mammary duct, and this may be accompanied by calcifications on MG, which can be confirmed as echogenic foci in some cases. As shown in Fig. 12, when DCIS lesions are present in relatively thick ducts, they can be observed as abnormalities of the ducts, and when these advance into thin ducts and lobules, they may be observed as hypoechoic areas [2].

In the present study, DCIS cases exhibiting abnormalities of the ducts were investigated. DCIS detected based on US findings of abnormalities of the ducts was 10.2% of cases in our study. According to data previously published by Izumori et al., it was 5.6% [13]. The data from Izumori et al. were from DCIS cases detected by US screening alone, but our data included cases with findings from mammography exams and subjective symptoms such as nipple discharge. Because these subjects were different, it was conjectured that the frequency of DCIS detected based on abnormalities of the duct was different.

By analyzing the details of the US findings of DCIS detected based on abnormalities of the duct in many institutions, it was possible to verify what had been empirically known. The distribution of abnormalities of the ducts was focal or segmental, which was a typical US appearance of DCIS with these abnormalities. The second US appearance was the presence of internal echoes in the dilated ducts. All cases were accompanied by solid echoes, and 40 (51.3%) of 78 cases were accompanied by echogenic foci suggesting microcalcifications on MG. In addition, solid echoes were not solitary and were continuous or multiple in 72 cases (92.4%), and the shape of solid echoes was broad-based and/or irregular, and appeared to be crawling on the wall in 38 cases (79.5%). Thus, these findings may serve as an index strongly suggesting DCIS.

Although duct abnormalities are included in “ASSOCIATED FEATURES” in ACR BI-RADS ATLAS, we emphasize that this concept is very important for understanding ultrasonographic characteristics of DCIS as an independent finding.

This study had some limitations. The study was designed as a retrospective study. Although the subjects had already been diagnosed with DCIS, differential diagnosis from benign diseases such as intraductal papilloma could not be described. In this study, only B-mode US was used, and color Doppler US and elastography were not evaluated. For this reason, this study was a multicenter study with various devices being used. The elastography devices, in particular, differed greatly from one device to another. Criteria for evaluation of non-mass abnormalities have not been established yet. It is important that JABTS establish diagnostic criteria in a timely manner when color Doppler and elastography are added. After diagnostic criteria have been established, research should be conducted by adding color Doppler and elastography to this study.

## Conclusion

This multicenter study was conducted in DCIS cases with duct abnormalities on ultrasound. The characteristics were as follows: (1) the distribution of ductal dilatation was focal or segmental, (2) solid parts were present in the dilated ducts, (3) the distribution of internal echoes was continuous or multiple, (4) the shape of solid echoes was broad-based and/or irregular, and (5) internal echoes were sometimes accompanied by echogenic foci. Accurate evaluation of these findings may be useful for diagnosing DCIS.
